# Predictive and Prognostic Value of DNA Damage Response Associated Kinases in Solid Tumors

**DOI:** 10.3389/fonc.2020.581217

**Published:** 2020-11-03

**Authors:** Mariam Gachechiladze, Josef Skarda, Katerina Bouchalova, Alex Soltermann, Markus Joerger

**Affiliations:** ^1^ Department of Clinical and Molecular Pathology, Institute of Translational and Molecular Medicine, Faculty of Medicine and Dentistry, Palacky University, Olomouc, Czechia; ^2^ Clinic of Pediatry, Faculty Hospital Olomouc, Olomouc, Czechia; ^3^ ADMED Pathology, Neuchâtel, Switzerland; ^4^ Department of Medical Oncology and Haematology, Cantonal Hospital, St. Gallen, Switzerland

**Keywords:** ATM, ATR, WEE1, inhibitor, prognostic

## Abstract

Dysfunctional DNA repair with subsequent genome instability and high mutational burden represents a major hallmark of cancer. In established malignant tumors, increased DNA repair capacity mediates resistance to DNA-damaging therapeutics, including cytotoxic drugs, radiotherapy, and selected small molecules including inhibitors of poly (ADP-ribose) polymerase (PARP), Ataxia Telangiectasia Mutated (ATM), ataxia telangiectasia and Rad3-related protein (ATR), and Wee1 kinase (Wee1). In addition, DNA repair deficiency is not only associated with sensitivity to selected anticancer drugs, but also with increased mutagenicity and increased neoantigen load on tumor cells, resulting in increased immunogenicity and improved response to CTLA4- or PD-(L)1 targeting monoclonal antibodies. DNA damage response (DDR) is composed of complex signalling pathways, including the sensing of the DNA damage, signal transduction, cellular response pathways to DNA damage, and activation of DNA repair. DNA double strand breaks (DSBs) are the most dangerous form of DNA damage. Tumor cells are characterised by frequent accumulation of DSBs caused by either endogenous replication stress or the impact of cancer treatment, most prominently chemotherapy and radiotherapy. Therefore, response of cancer cells to DSBs represents a crucial mechanism for how tumors respond to systemic treatment or radiotherapy, and how resistance develops. Ample clinical evidence supports the importance of DDR associated kinases as predictive and prognostic biomarkers in cancer patients. The ATM-CHK2 and ATR-CHK1-WEE1 pathways initiate DNA DSB repair. In the current review, we focus on major DDR associated kinases including ATM, ATR, CHK1, CHK2, and WEE1, and discuss their potential prognostic and predictive value in solid malignancies.

## DNA Damage Response 

DNA is constantly damaged by different exogenous and endogenous factors. DNA lesions vary from simple base changes to strand breaks. There are two major types of DNA strand lesions, i.e. single strand breaks (SSBs) and double strand breaks (DSBs). SSBs are indirectly caused by alkylating agents, UV light, and PARP inhibitors, amongst other insulting agents, irradiation can directly cause SSBs and DSBs. In addition, SSBs can transform into DSBs when replication forks stall and collapse. Therefore, cellular response to DSBs represent the major mechanism affecting malignant transformation and treatment response. There are two major pathways for DNA DSB reparation, i.e. high-fidelity homologous recombination (HR) and error-prone non-homologous end joining (NHEJ). Cellular response mechanisms to DNA DSB have outstandingly been discussed elsewhere (1–6) and is beyond the scope of this review. Here, we will focus on potential clinical applications of major DDR kinases activated by DNA DSBs. Key DDR-signalling pathways in mammalian cells include the protein kinases ataxia telangiectasia mutated (ATM) and ataxia telangiectasia and Rad3-related (ATR). ATM is directly activated by DNA DSB’s, while ATR is activated by RPA-coated single strand DNA (ssDNA) later in the DSB signalling process (7–9). In addition, ATR can be activated by RPA-coated single strand DNA (ssDNA) in the absence of DSBs. Activation of ATM and ATR result in subsequent activation of CHK2 and CHK1, respectively, which, together with ATM and ATR, result in strong inhibition of cyclin-dependent kinase (CDK) activity by various mechanisms including activation of p53 transcription factor (8, 10, 11). ATM/ATR signalling initiates DNA repair by transcriptional and post-transcriptional regulation of DNA-repair-proteins as well as recruitment of repair factors to damaged DNA, and by activating DNA-repair proteins by post-translational modifications including phosphorylation, acetylation, ubiquitination, or sumoylation (12). One of the major effector DDR-associated proteins is the WEE1 kinase. WEE1 is activated through phosphorylation by CHK1 and promotes cell-cycle arrest and DNA damage repair (13). WEE1 controls CDK1 and CDK2 activity during the G2-M and S phase, respectively. WEE1 causes the suppression of replication origins firing, promotes homologous recombination (HR), and prevents excessive resection of stalled replication forks (14, 15). It has been shown that inhibition of WEE1 impairs HR-mediated DNA repair through activation of CDK1 and subsequent inhibitory phosphorylation of BRCA2 (15). Additionally, inhibition of WEE1 causes the persistence of ɣH2AX foci, a prominent marker of DNA DSBs, and inhibits the formation of RAD51 foci as a surrogate marker of HR repair (16). In their pioneering work Bartkova et al., have described that activation of DDR serves as an anti- cancer barrier at earlier stages of malignancy (17).

**Figure 1:**
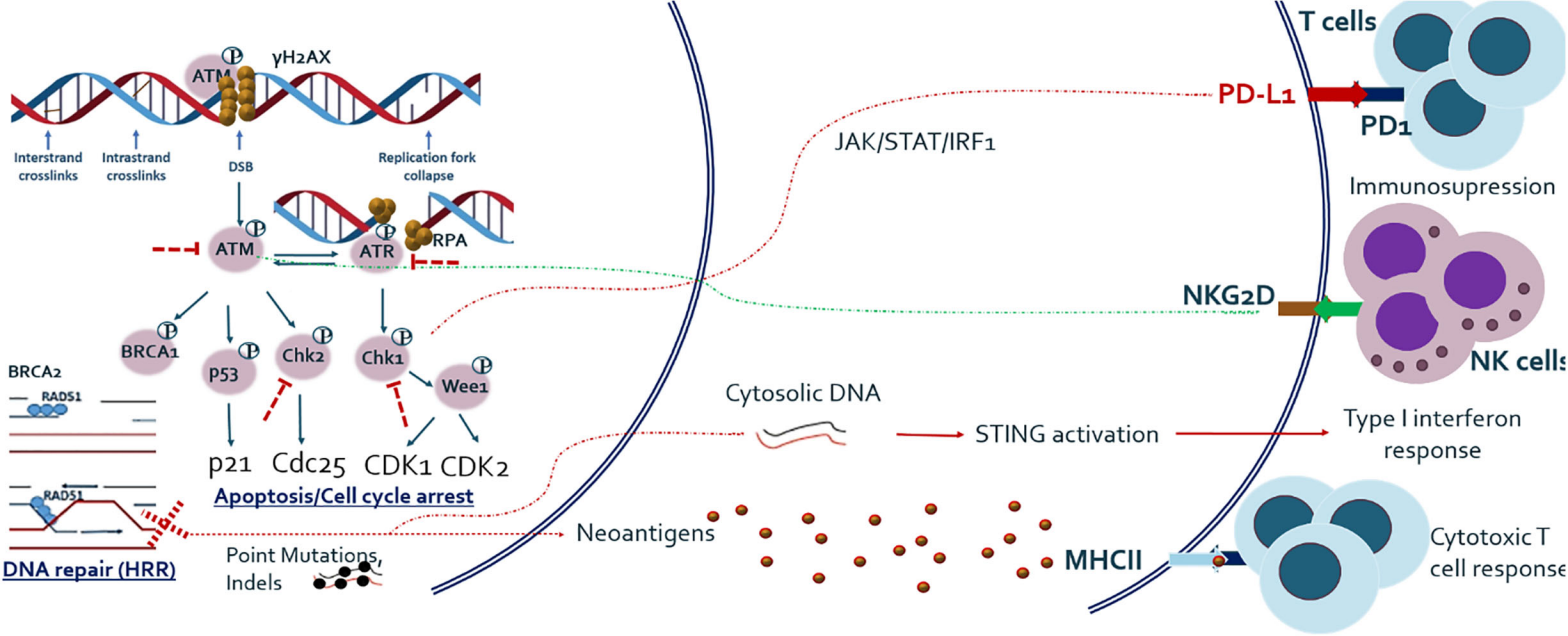
Outline of major DNA DSB repair pathway and its relationship with host anti-tumor immune response. Studies have shown that DNA damage response and repair (DDR/R) have a double effect on host immunity, either immune activating or immunosuppressive. DNA repair and especially HRR deficiency causes one hand the increased load of mutations and hence neo-antigens, which activate adaptive immune system and on the other hand the release of cytoplasmic DNA which activates innate immune system. Major DDR kinase ATM is also shown to be directly implicated in the upregulation of NKG2D ligand which mediates NK cell dependent killing of tumor cells. ATM/ATR/CHK1 axis also upregulates PD-L1 expression after DNA damage, via JAK/STAT/IRF1 pathway.

In addition to cell cycle arrest, DNA repair, and apoptosis, recent studies have shown that DDR pathway alterations are related to various types of host immune response, which has extensively been reviewed elsewhere (18, 19). In summary, deficiency of DNA DSB repair and particularly HRR is related to increased tumor mutational burden (TMB) and neoantigen load on tumor cell surface, which subsequently activates adaptive immune response (20). DSB repair deficiency has also been shown to be associated with release of self-DNA in the cytoplasm, which is recognized by innate immune sensors and subsequently activates the STING-mediated type I interferon pathway. In addition, the ATM/ATR/CHK1 pathway has been shown to be essential for upregulation of PD-L1 on cancer cells after treatment with DNA-damaging agents (21). It has also been demonstrated that NK cell ligand NKG2D is upregulated in response to DNA damage and ATM is directly involved in its upregulation (22). The overview of basic research findings regarding cross-talk between DDR and immune system is given in **Figure 1**.

Major DNA damage response and repair (DDR/R) kinases have been the subject of numerous pre-clinical and clinical investigations as markers of standard of care treatments including radiotherapy, chemotherapy, and the newer small molecule inhibitors of PARP, ATM, ATR, CHEK1/2, and WEE1. More recently, DDR/R proteins attracted new interest as potential markers of response to immunotherapy in cancer patients. In this review, we will discuss pre-clinical and clinical data related to major DDR/R proteins as potential prognostic and/or predictive biomarkers in solid malignancies.

## Prognostic and Predictive Value of Major DDR Proteins

### ATM

According to The Cancer Genome Atlas (TCGA) pan cancer studies (23), ATM somatic mutations are most frequently found in endometrial cancer (~18.7%), followed by bladder cancer (~12.9%) and colorectal cancer (~11.8%). ATM somatic mutations are also frequent in melanoma (~9.2%) and lung adenocarcinoma (~8.1%). As for ATM deep deletions (deep loss, possibly homozygous deletion as defined by cBiopostal), it is most frequently found in cervical cancer and melanoma, representing 2.4 and 2.3% of all cases, respectively. ATM deep deletions are very rare in other cancer types (**Supplementary Figure 1**) (23). ATM mutations may result in chemoresistance, serve as a poor prognostic factor, and may also be exploited by existing or emerging targeted therapies leveraging on the principle of synthetic lethality [Reviewed by Choi et al. (24)]. Some studies also looked at loss of total ATM protein by immunohistochemistry (IHC), which varies from 5 to 41% across different tumor types. Sundar et al. demonstrated total ATM loss in 8% of colorectal carcinoma samples, (25) whilst it was found in 14% of prostate carcinoma cases by Antonarakis et al., (26) and ATM loss reached 41% in lung adenocarcinoma cases according to Villaruz et al. (27). Although the mechanism is not completely clear so far, pre-clinical data support synthetic lethality of ATM-deficient cells towards ATR inhibition, and clinical trials are underway to test ATR inhibitors in patients with ATM-deficient tumors.

Preclinical studies have demonstrated an important role of ATM in radiosensitivity. Ayars et al. showed that ATM-deficient pancreatic cancer cell lines do not show specific sensitivity to selected chemotherapeutic agents, but they were highly sensitive to irradiation (28). Another study showed that elevated ATM expression, induced by DAB2IP-knockdown [disabled homolog 2 interactive protein (DAB2IP)—a tumor suppressor gene] might represent a key event in bladder cancer radioresistance (29). Li et al., demonstrated that silencing of ATM by siRNA significantly improved radiosensitivity of glioma stem cells both in vitro and in vivo (30). In addition, the treatment with ATM inhibitors increased the radiosensitivity of glioma cells (31) and glioblastoma stem cells (32) in vitro. Concordantly, clonogenic survival analysis revealed inhibition of ATM to lead to pronounced radio-sensitivity of cervical cancer cells. The colorectal cancer cell line SK-CO-1 lacking detectable ATM protein expression has been shown to be sensitive to the PARP inhibitor olaparib, similar to HCT116 cells following siRNA-mediated depletion of ATM (33). Moreover, HCT116 cells are sensitive to olaparib in combination with the ATM inhibitor KU55933, and sensitivity is further enhanced by deletion of p53 (33). The study by Subhash et al. evaluated ATM-induced synthetic lethality and its role in the sensitization of gastric cancer cells to inhibitors of PARP (veliparib) and TOP1 (irinotecan). Cells with high ATM expression were characterized by reduced sensitivity to single-agent veliparib or irinotecan, but they kept sensitivity to the combination of veliparib and irinotecan (34), which reflects the concept of synthetic lethality. Recently, Zhang and colleagues have demonstrated that inhibition of ATM increases interferon signaling and sensitizes pancreatic cancer to immune checkpoint inhibition (35). Particularly, inhibition of ATM increased tumoral T1IFN expression in a TBK1- and SRC-dependent manner. The combination of ATM inhibition with irradiation further enhanced TBK1 activity, T1IFN production and antigen presentation. In addition, ATM silencing increased PD-L1 expression and increased the sensitivity of pancreatic cancer to immune checkpoint inhibition in association with increased tumoral CD8+ T cells and established immune memory (35).

In line with the in vitro data, clinical studies also show that ATM has predictive as well as prognostic value in patients with advanced solid tumors. Analysis of tissue microarrays from 165 non-small cell lung cancer (NSCLC) patients using quantitative fluorescence immunohistochemistry identified ATM loss in 21.8% of patients (36). ATM loss was significantly and independently associated with poor overall survival (OS) in stage II/III patients receiving potentially curative treatment including surgery and adjuvant chemotherapy (36). Similarly, a study in breast cancer patients showed that in patients with both hormone receptor-positive (HR+) and receptor-negative (HR-) breast cancer, low ATM expression by IHC was associated with HR negativity and poor OS. Multivariate analysis showed that low ATM was associated with poor OS independent of tumor size and lymph node status, but only in HR- breast cancer (37). Ho and colleagues studied cervical cancer patients undergoing chemo-radiotherapy (38). In these patients, five-year progression-free survival (PFS) was significantly lower in ATM low-expressing tumors compared to ATM high-expressing tumors (35 vs. 58%, p = 0.044) (38). On the contrary, Ronchetti et al. found that the combination of two biomarkers (γ-H2AX^high^/pATM^high^) was associated with worse PFS (multivariate Cox HR 2.23, 95%CI: 1.47–3.40) and OS (multivariate Cox: HR 2.07, 95%CI: 1.20–3.58) in patients with advanced gastric cancer undergoing first-line platinum-based chemotherapy (39). In lung adenocarcinoma patients, Villaruz et al. found ATM loss by IHC to be present in 41% of patients, but there was no significant relationship between ATM loss and OS. However, the information about disease stage and patient treatment was not given in the manuscript (27). Low ATM was also associated with advanced TNM stage and poor 5-year OS in patients with colorectal cancer (CRC) (40). Analysis of ATM protein expression in 908 stage II/III CRC patients from the VICTOR randomised controlled trial comparing the effect of adjuvant rofecoxib against placebo in reducing recurrence in patients who had undergone tumor resection showed that low ATM expression compared to normal mucosa was independently associated with poor disease-free survival (HR = 1.67, 95% CI 1.11–2.50, p = 0.015) (41). The expression of ATM and TP53 was determined by IHC in 397 surgically resected pancreatic ductal adenocarcinoma patients and a second set of 159 resected pancreatic cancer patients and 21 patients receiving neoadjuvant systemic treatment (42). Fifty out of 397 patients (12.8%) showed tumoral ATM loss, and the latter was associated with poor OS in patients with normal TP53 expression (p = 0.019). Seventeen out of 159 patients (10.7%) from the second set had tumoral ATM loss, and ATM loss in combination with normal TP53 was again associated with poor OS (P = 0.01). Multivariate analysis found that tumoral ATM loss/normal TP53 was an independent risk factor for poor OS [HR = 2.61; 95%(CI), 1.27–5.37; P = 0.009]. Of 21 cancers examined after neoadjuvant chemo-radiotherapy, one patient had tumoral ATM loss and no histologic evidence of tumor response at the time of surgery (42). In 52 breast cancer samples, low ATM levels compared to normal breast tissue were associated with high tumor grade, while ATM loss was associated with distant metastasis (P < 0.001), worse disease free survival (DFS) (P < 0.001) and cancer-specific survival (CSS) (P < 0.001). Multivariate analysis indicated ATM protein expression to be an independent favorable prognostic marker for DFS (P = 0.001, HR = 0.579) and CSS (P = 0.001, HR = 0.554) (43). ATM gene loss has also been found to be a frequent event in HNSCC, but it lacked prognostic or predictive impact in this group of HNSCC patients undergoing chemo-radiotherapy according to Lim et al. (44). In patients with nasopharyngeal cancer (NPC) receiving concurrent chemoradiotherapy, high ATM protein expression by quantitative fluorescent immunohistochemistry was associated with poor OS [hazard ratio (HR), 2.83; 95% CI, 1.01–7.94; p = .049] (45). In endometrial cancer, combined positive expression of ATM and p53 or FANCD2 was associated with poor 5-year relapse-free survival (RFS) as compared to negative ATM/p53 or FANCD2 (68% versus 80.3%, p = 0.0241) (46). Roossink at al tested whether tumoral ATM expression was predictive for treatment outcome with adjuvant chemo-radiothrerapy in 375 patients with advanced cervical cancer (47). ATM expression was useful to stratify the low Ki67 group into prognostic subgroups in patients with breast cancer. Specifically, patients with low Ki67 were characterized by smaller tumors and a lack of lymph node metastases. In this group of patients, those with high ATM expression had a better OS compared to those with low ATM, with estimated survival rates of 96 and 89% at 15 years, respectively (p = 0.04). Similarly, patients with small tumors and low Ki67, 1-3 positive lymph nodes and high ATM expression had a significantly higher OS compared to patients with low ATM expression with estimated 15-year OS rates of 88 and 46%, respectively (p = 0.03). Multivariate analysis indicated that the combination of high ATM and low Ki67 is prognostic for improved OS, independent of tumor size, grade, and lymph node status (p = 0.02) (48). One study also examined the predictive and prognostic role of phosphorylated (p-)ATM expression in cervical cancer and it was found that high levels of p-ATM was associated with poor loco-regional tumor control (HR = 1.817, p = 0.006). Furthermore, high levels of p-ATM predicted poor disease-specific survival (p = 0.038, HR = 1.418) (47). Summary of clinical studies examining the prognostic and/or predictive value of ATM loss in solid malignancies is given in **Table 1**.

**Table 1:** Studies examining prognostic and/or predictive value of ATM in solid malignancies.

Author	Year	Cancer type	Sample Size	Study Intervention	Methodology for ATM detection	Study endpoint	ATM loss/low (%)	Outcome	RR/HR and 95%CI	*P Value*
**Beggs et al. (** 41 **)**	2012	Colorectal CA	908	Adjuvant CHT	IHC	DFS	N.A.	Poor DFS	DFS (HR = 1.67, 95% CI 1.11–2.50)	*p = 0.015*
**Lim et al. (** 44 **)**	2012	HNSCC	73	Adjuvant CHT/RT	IF	OS	60.30%	Not prognostic	OS [HR = 1.16, 95% (CI), 0.59–2.29]	*p = 0.67*
**Roossink et al. (** 47 **)**	2012	Cervical CA	375	Adjuvat RT or CHT/RT	IHC	DFS	N.A.	Better DFS	DFS (HR = 1.418)	*p = 0.038*
**Kim et al. (** 42 **)**	2014	Endometrial CA	357	Neoadjuvant CHT	IHC	OS	N.A.	Poor OS	OS [HR: 2.61; 95%(CI), 1.27–5.37]	*P = 0.009*
**Kim et al. (** 42 **)**	2014	Pancreatic ADC	396* and 159**	Surgery	IHC	OS	12.8* and 10.7%**	Poor OS	OS [HR = 3.199, 95% (CI), 1.341–7.627]	*p = 0.009*
**Bueno et al. (** 43 **)**	2014	Breast CA	968	N.A.	IHC	OS, DFS	N.A.	Poor DFS	DFS [HR: 0.579 (0.421–0.797)]	*p = 0.001*
**Feng et al. (** 37 **)**	2015	HNBC	168	Surgery with or without Adjuvant CHT/RT	IF	OS	N.A.	Poor OS	OS [HR: 7.43, 95%CI: (2.95–18.76)]	*p < 0.001*
**Villaruz et al. (** 27 **)**	2016	Lung ADC	147	N.A.	IHC	OS	41%	Not prognostic	N.A.	*N.A.*
**Ko et al. (** 45 **)**	2016	Nasopharingeal CA	58	Chemoratiotherapy	IHC	OS	N.A.	Better OS	OS (HR: 0.44; 95% CI, 0.20–0.94)	*p = 0.033*
**Petersen et al. (** 36 **)**	2017	NSCLC	165	Surgery with or without Adjuvant CHT	IF	OS, DFS	21.80%	Poor OS and DFS	OS (HR: 5.09, 95% CI: 2.07–12.52); DFS (HR: 4.75, 95% CI: 2.02–11.17)	*p < 0.001*
**Ho et al. (** 38 **)**	2017	Cervical CA	166	Surgery with or without Adjuvant CHT	IF	PFS	N.A.	Poor PFS	PFS (HR: 1.8, 95%CI: 1.0–3.4)	*p = 0.047*
**Sundar et al. (** 25 **)**	2018	Colorectal CA	223	Surgery with or without Adjuvant CHT	IHC	OS	8%	Better OS	OS (HR: 2.52, 95% CI: 1.00–6.37)	*P = 0.05*
**Antonarakis et al. (** 26 **)**	2019	Prostate CA	> 1,000	N.A.	IHC	N.A.	14%	N.A.	N.A.	*N.A.*

*training set; **validation set; CA, carcinoma; HNSCC, head and neck squamous-cell carcinoma; HNBC, hormone negative breast cancer; ADC, adenocarcinoma; NSCLC, non-small cell lung carcinoma; IHC, immunohistochemistry; IF, immunofluorescence; OS, overall survival; PFS, progression free survival; DFS, disease free survival; CHT, chemotherapy; RT, radiotherapy; HR, hazard ratio; RR, relative risk.

### ATR

ATR mutations are most frequently found in endometrial carcinoma (~12.1%), followed by melanoma (~11.7%) and bladder cancer (~7.5%). ATR deep deletions are very rare and found in a minority of mesothelioma (~1.2%) and squamous-cell lung cancer (~0.4%). A further common genetic alteration is ATR amplification that is found in about 10.1% of lung squamous-cell carcinoma, ~6.6% of esophageal cancer, ~5.7% of head and neck carcinomas and a minority of other cancer cases (**Supplementary Figure 2**) (23).

In endometrial cancer, Zighelboim et al. have found ATR mutations to be associated with microsatellite instability (49). In the study by Zighelboim et al., multivariate analysis revealed a significant association between ATR mutations and poor OS (HR = 3.88; 95% CI, 1.64 to 9.18; P = .002) as well as poor DFS (HR = 4.29; 95% CI, 1.48 to 12.45; P = .007) (49). Another study by the same group however found no significant association between ATR mutations and OS (HR 1.16; 95% CI, 0.58–2.32; p=0.68) or DFS (HR 0.61; 95%CI, 0.25–1.50; p=0.28) in endometrial cancer (49). Li et al. showed that inhibition of the ATR-CHK1 pathway using the small molecule inhibitor WYC0209 sensitized bladder cancer cells to cisplatin (50). Similarly, inhibition of ATR by siRNA significantly increased the level of cisplatin-DNA adducts in bladder cancer cells (51). Sun et al. showed that inhibition of ATR downregulates PD-L1 and sensitizes various cancer cells to CD8+ T cell-mediated killing, including lung A549, cervical Hela and breast MDA-MB-231 cells (52). The study further showed that DNA-damaging agents result in an induction of PD-L1 expression on tumor cells, and this was prevented by depletion or pharmacological inhibition of ATR. Inhibition of ATR resulted in destabilization of PD-L1 in a proteasome-dependent manner, attenuation of the interaction of PD-L1/PD-1 and sensitization of cancer cells to T-cell mediated killing (52).

In clinical studies, low expression of cytoplasmic p-ATR was significantly associated with advanced stage, serous histology, and high preoperative serum CA125 concentrations in patients with epithelial ovarian cancer (53). In the study by Lee et al., univariate survival analysis revealed that low cytoplasmic p-ATR expression was significantly associated with poor DFS (HR = 2.2, 95%CI, 1.2–4.2, p < 0.05) and poor OS (HR = 2.3, 95%CI, 1.2–6.1, p < 0.05) (53). In the study of Abdel-Fatah et al., high ATR expression levels were associated with high tumor stage (*p* = 0.036), high tumour grade (*p* < 0.001), high mitotic index (*p* < 0.001), polymorphism (*p* < 0.001), and lymphovascular invasion (*p* = 0.009) in patients with breast cancer (54). Summary of clinical studies examining the prognostic and/or predictive value of ATR loss in solid malignancies is given in **Table 2**.

**Table 2:** Studies examining prognostic and/or predictive value of ATR, CHK1, CHK2, and WEE1 in solid malignancies.

Author	Year	Cancer type	Sample Size	Study Intervention	Methodology for marker detection	Study endpoint	ATR Loss/Low (%)	Outcome	RR/HR and 95%CI	*P value*
**Abdel-Fatah et al. (** 54 **)**	2014	Ovarian CA	194	Surgery,Adjuvant CHT	IHC	OS, PFS	67.80%	Poor OS and PFS	N.A.	*OS (p = 0.001), PFS (p = 0.008)*
**Lee et al. (** 53 **)**	2015	Ovarian CA	100	Surgery,Adjuvant CHT	IHC	OS, DFS	45/62%	Poor OS and DFS	OS (HR = 8.9, 95% CI = 2.6–30.0)DFS (HR = 6.5, 95% CI = 2.5–16.8)	*p < 0.001*
							**CHK1 Loss/Low**			
**Al-Kaabi et al. (** 55 **)**	2015	Breast CA	1200	Suegery, Adjuvant CHT	IHC	OS	50% nuclear, 6% cytoplasmic	Nuclear: better OS Cytoplasmic: poor OS	N.A.	*p = 0.014 for Nuclear and p = 0.027 for cytoplasmic*
							**CHK2 Loss/Low**			
**Kilpivaara et al. (** 56 **)**	2005	Breast CA	389	N.A.	IHC	OS	21.10%	not prognostic	N.A.	*N.A.*
**Lee et al. (** 57 **)**	2014	Gastric CA	524* and 394**	Surgery,Adjuvant CHT	IHC	PFS	14.1* and 12.2%**	Poor PFS	PFS [HR = 1.390, 95%CI (1.003–1.927)]	*p < 0.001*
							**Wee1 High**			
**Yoshida et al. (** 58 **)**	2004	NSCLC	79	N.A.	IHC	DFS	34.20%	Poor DFS	PFS [HR = 9.169 95%CI (1.057–79.526)]	*p = 0.0444*
**Magnussen et al. (** 59 **)**	2012	Melanoma	108	N.A.	IHC	DFS	20%	Poor DFS	N.A.	*p =0.005*
**Slipicevic et al. (** 60 **)**	2014	Ovarian Ca	287	Neoadjuvant CHT	IHC	OS	N.A.	Poor OS	N.A.	*p =0.004*
**Music et al. (** 61 **)**	2016	Glioma	235	N.A.	IHC	OS	24.5%	Better OS	OS (HR = 0.60)	*p = 0.003*
**Ge et al. (** 62 **)**	2017	Colorectal CA	102	N.A.	IHC	OS, PFS	28.40%	Poor OS	OS (HR = 3.339); PFS (N.A.)	*P = 0.039*

*Training set; **Validation set; CA, carcinoma; NSCLC, Non-small cell lung cancer; IHC, immunohistochemistry; OS, overall survival; PFS, progression free survival; DFS, disease free survival; CHT, chemotherapy; HR, hazard ratio; RR, relative risk.

### CHK1 and CHK2

According to TCGA pan cancer studies (23), the most frequent genetic alterations of CHEK1 is deep deletion, which is most commonly found in testicular germ cell tumors (~8.7%) and uveal melanoma (5%). CHEK1 point mutations are most frequently found in endometrial cancer (~3.8%) and in lung squamous-cell carcinoma (~2.1). Overall, CHEK1 genetic alterations in various cancer types are rare (**Supplementary Figure 3**). With regard to CHEK2, most frequent genetic alterations are point mutations, which are seen in 6.4% of endometrial cancer. Generally, multiple different kinds of CHK2 genetic alterations are found over various cancer types, including deep deletions, amplifications, gene fusions and rarely also multiple gene alterations in the same patient (**Supplementary Figure 4**) (23).

Kilpivaara et al. investigated CHK2 protein expression by immunohistochemistry in tissue microarrays from 611 breast cancer patients and the presence of CHK2 germline mutations in 1,297 breast cancer patients. The authors found CHK2 protein expression to be decreased in 21.1% of breast cancer patients, whilst germline mutations were detectable in 2.5% of patients. Tumors with low CHK2 expression were characterized by large primary tumors (pT3-4, p=0.002) compared to tumors with normal staining (56).

The expression level of phosphorylated Chk1 (p-Chk1) was higher in radio-resistant lung cancer cell lines compared to radio-sensitive cell lines (63). Treatment with the small molecule CHK1 inhibitor AZD7762 significantly sensitized both radio-resistant and radio-sensitive cells to irradiation (63). In the same study, investigators observed a strong inverse association between the expression of p-CHK1 by IHC and PFS (63). Lee et al. found CHK2 protein loss in 14.1% of patients with advanced gastric cancer and CHK2 loss was significantly associated with advanced TNM stage and poor DFS (HR = 1.970 95%CI:1.245–3.116, p < 0.001). Multivariate analysis found loss of CHK2 to be an independent prognostic factor for poor DFS (HR = 1.390, 95%CI = 1.003–1.927, p < 0.001) (57). Expression of CHK2 and pCHK2 was found to be roughly 50% lower in around 50% of colorectal carcinomas (64). Quantitative studies revealed significantly lower p-CHK2 expression in early stages of colorectal carcinomas compared to advanced stages. Furthermore, high p-CHK2 expression was associated with high nodal status (64). Overall, the data from Stawinska et al. suggest p-CHK2 expression to be associated with worse prognosis in patients with colorectal cancer (64). Honrado and colleagues studied CHK2 protein expression and its correlation with somatic BRCA mutations in 103 familial and 104 sporadic breast cancer patients (65). The authors found CHK2 protein to be more frequently expressed in patients with rare tumors and somatic mutations of BRCA1 or BRCA2 as compared to BRCA1 or BRCA2 wild type tumors (78.4 versus 39.3%, p > 0.05) (65). Low CHK2 expression in breast cancer patients was associated with high T stage (pT3-4; p = 0.002) compared to tumors with moderate staining. However, no correlation was seen between CHK2 IHC status and hormone receptor status, histology, lymph node status, and clinical outcome in this group of breast cancer patients (65). Among 58 patients with locally advanced bladder cancer, immunohistochemical analysis revealed low CHK2 protein expression in 6 (10.3%) cases compared to corresponding normal bladder epithelium (66). Zhang et al. reported low or absent CHK2 expression in non-small-cell lung carcinomas to result from hypermethylation of the CHK2 gene promoter, leading to subsequent silencing of CHK2 gene transcription (67). Recently, Sato et al. demonstrated PDL1 expression to be upregulated in cancer cells in response to DSBs under genotoxic stress, such as radiotherapy or PARP inhibition. Using siRNA library screen targeting DNA repair genes, the authors showed that PD-L1 induction in this setting was dependent on the ATM/ATR/Chk1 pathway (68). Summary of clinical studies examining the prognostic and/or predictive value of CHK1 and 2 loss in solid malignancies is given in **Table 2**.

### WEE1

Genetic alterations of WEE1 in solid tumors are very rare as shown by the analysis from TCGA pan-cancer studies (23). WEE1 point mutations are most frequently found in endometrial carcinoma (~3.2%) followed by bladder cancer (~2.2%). WEE1 deep deletions have been found in low-grade glioma (~2.71%). Mutations, deletions, and other genetic alterations of WEE1 in other solid tumors are very rare (**Supplementary Figure 5**) (23).

Inhibition of WEE1 by the selective WEE1 inhibitor MK-1775 resulted in anti-tumor effects in several preclinical tumor models including colon cancer, suggesting WEE1 as a potential therapeutic target for anticancer treatment (62). Increased WEE1 mRNA expression has been observed in numerous solid tumor entities including hepatocellular carcinoma and melanoma (62). The analysis of WEE1 mRNA expression in 43 cases of colorectal carcinomas showed significantly higher WEE1 mRNA expression in tumor tissue compared to adjacent healthy tissue, and high WEE1 mRNA expression was significantly associated with high tumor stage (62). Analysis of WEE1 immunohistochemical expression in 102 colorectal carcinoma patients revealed high WEE1 expression to be associated with both poor OS (P = 0.018) and poor DFS (P = 0.039). Multivariate analysis showed high WEE1 IHC expression to be an independent negative prognostic factor in this group of patients (HR, 2.392; P = 0.023) (62). Nuclear expression of WEE1 was detected in 229 out of 258 (89%) colorectal carcinomas according to Egeland et al. (2016) (69). WEE1 staining was associated with low pT stage, but no other significant association with demographic or histopathological parameters was found. Inhibition of Wee1 expression using siRNA or treatment with the specific Wee1 inhibitor MK-1775 resulted in reduced expression of the metastasis-promoting protein S100A4, but no association between WEE1 and S100A4 was found in the patient samples (69). Music et al. found higher WEE1 IHC expression in tumor tissue from glioblastoma multiforme (GBM) samples compared to grade II or III glioma, and high WEE1 IHC expression was independently associated with improved OS in GBM patients (HR 0.60, p = 0.003) (61). Slipicevic and colleagues compared WEE1 IHC expression in cytology specimens from the effusions of ovarian cancer patients before and after platinum-based combination chemotherapy (60). WEE1 expression was higher in post platinum-based chemotherapy specimens compared to pre-chemotherapy specimens (p = 0.002). Survival analysis showed WEE1 expression in 109 post-chemotherapy patients to be significantly associated with poor OS (p = 0.004), and this finding resisted multivariate Cox regression analysis (p = 0.003) (60). This data suggest WEE1 to play a potential role in the development of platinum resistance in ovarian carcinoma. In addition, in vitro experiments showed that Wee1 silencing in SKOV3 and OVCAR8 ovarian cancer cells significantly reduced proliferation (60). In vulvar squamous-cell carcinomas, WEE1 IHC overexpression was significantly associated with high N-stage and poor histological differentiation. siRNA-mediated silencing of WEE1 led to modest reduction of viability in corresponding vulvar cancer cell lines. However, a marked increase in DNA damage as assessed by augmented levels of γ-H2AX was observed in cell lines in the absence of Wee1 (70). Similarly, WEE1 expression was significantly associated with high tumor stage (p = 0.001), ulceration (p = 0.005), and poor disease-free survival (p = 0.008) in 108 melanoma patients (59). Transfections using siWEE1 in metastatic melanoma cell lines (WM239, WM45.1, LOX) further support the hypothesis of a potential tumor-promoting role of WEE1 in melanoma (59). In osteosarcoma patients, WEE1 was found to be significantly upregulated by immunohistochemistry compared to various normal tissues (71). In the presence of the WEE1-inhibitor PD0166285, irradiated cells failed to repair their damaged DNA, resulting in significant activation of caspase signalling (71). In 79 unselected NSCLC patients lacking expression of WEE1, Yoshida et al. found a higher recurrence rate (P = 0.0084), a poorer prognosis (P = 0.0457), and a higher Ki67 index and PCNA-labelling index values. Multivariate analysis suggested WEE1 to be a significant independent prognostic factor in this group of patients [HR = 9.169 95%CI(1.057–79.526) P = 0.0444] (58). Summary of clinical studies examining the prognostic and/or predictive value of Wee1 overexpression in solid malignancies is given in **Table 2**.

## Concluding Remarks

Ample clinical data demonstrate the prognostic and predictive value of major DDR proteins with regards to radiotherapy, chemotherapy or molecularly-targeted systemic treatment in several solid tumor entities. More recently, accumulating data are showing DDR proteins to be implicated in anti-tumor immune response, why DDR proteins may also be considered as potential biomarkers for immune checkpoint inhibitor treatment in the future. Accordingly, we expect genetic alterations and/or immunohistochemical expression of major DDR proteins including ATM, ATR, CHK1/2 and WEE1 to play an increasing role in tailoring anticancer treatment, including systemic treatment with PD(L)1-targeting monoclonal antibodies (checkpoint inhibitors), inhibitors of ATR, CHK1 or WEE1. Additional research is justified to validate DDR genetic alterations or immunohistochemical expression for their use in solid cancer patients.

## Author Contributions

All authors contributed equally in the writing of the review article. All authors contributed to the article and approved the submitted version.

## Funding

The publication is supported by the internal grant from Palacký University (LF_2019_004) and by the European Regional Development Fund – Project ENOCH (No. CZ.02.1.01/0.0/0.0/16_019/0000868) to M.G. and Foundation for Applied Cancer Research to AS.

## Conflict of Interest

The authors declare that the research was conducted in the absence of any commercial or financial relationships that could be construed as a potential conflict of interest.
