# Supplementary figures and images for: Predictive and Prognostic Value of DNA Damage Response Associated Kinases in Solid Tumors
Source: Front Oncol. 2020 Nov 3;10:581217. doi: 10.3389/fonc.2020.581217 (PMC7670868; doi:10.3389/fonc.2020.581217)

Figure S1,  
ATM

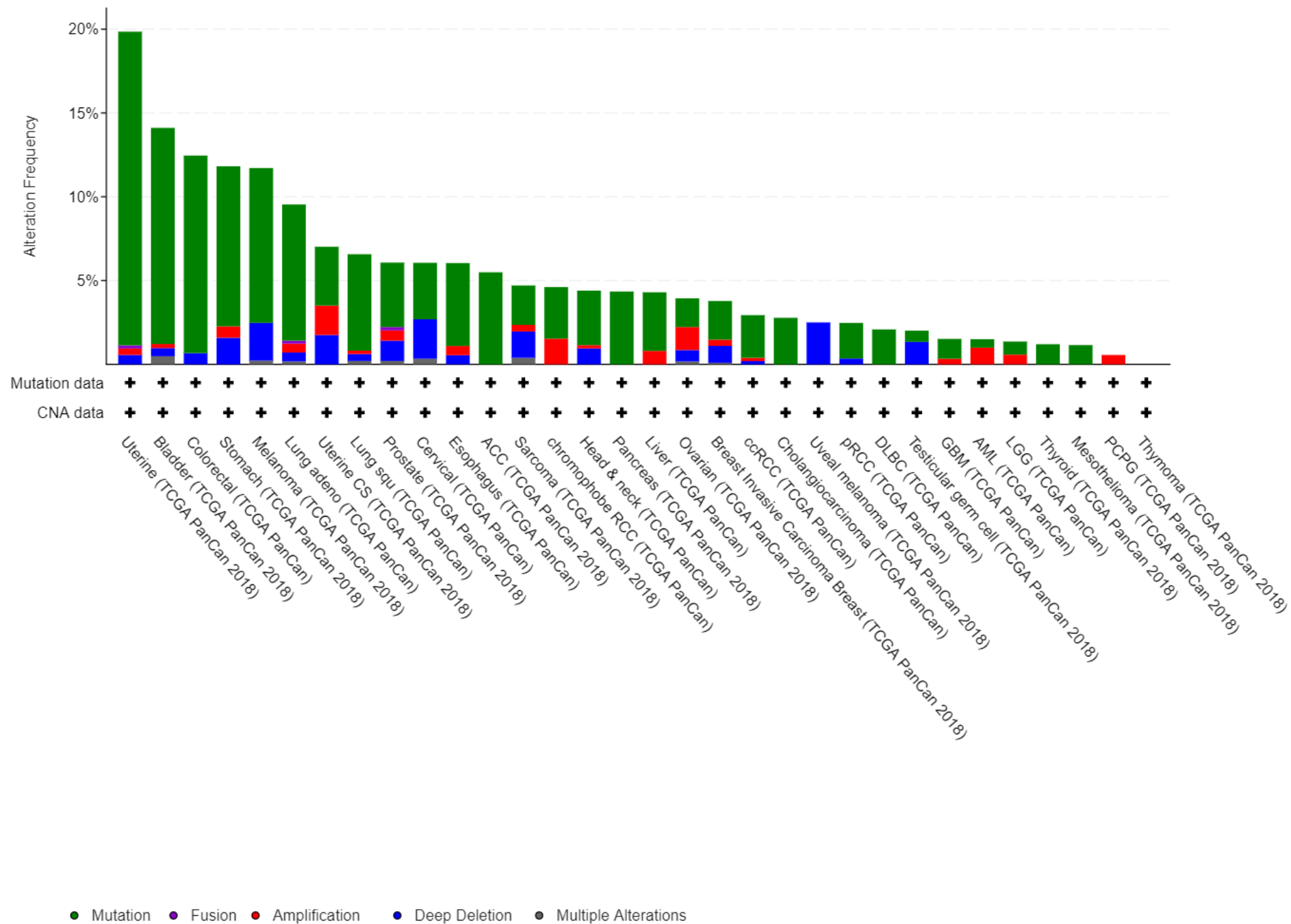

Figure S2,  
ATR

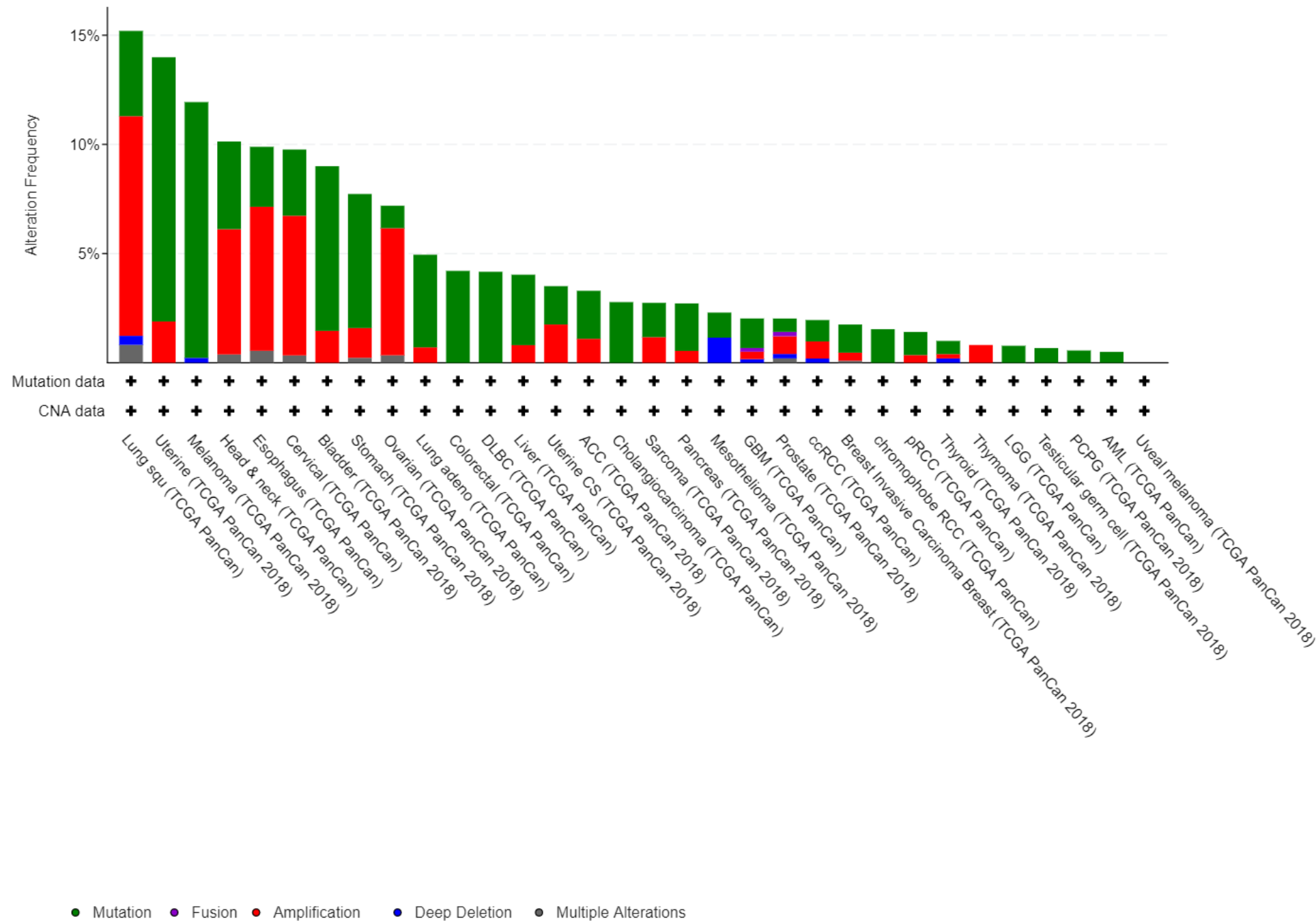

Figure S3,  
CHK1

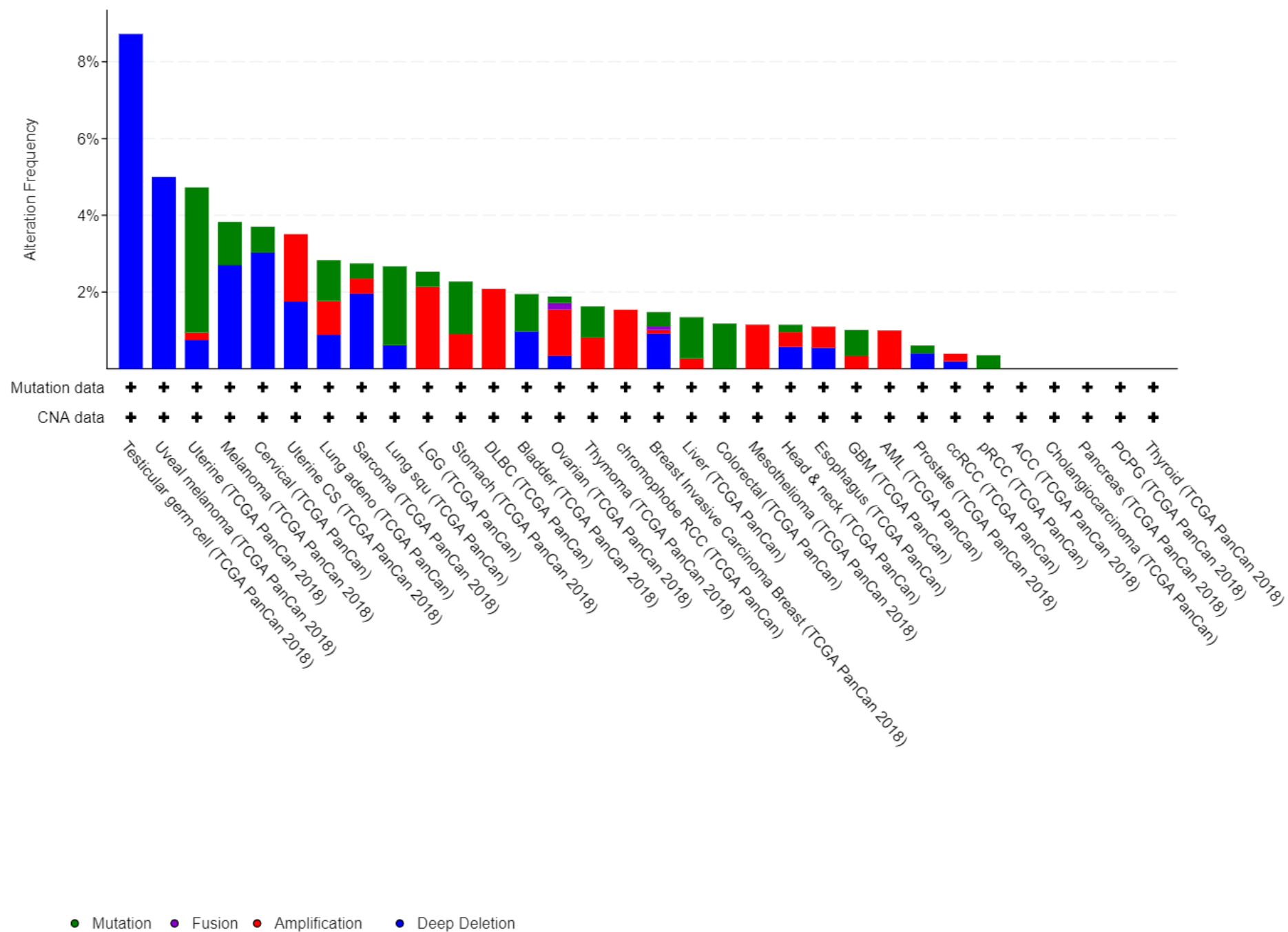

Figure S4,  
CHK2

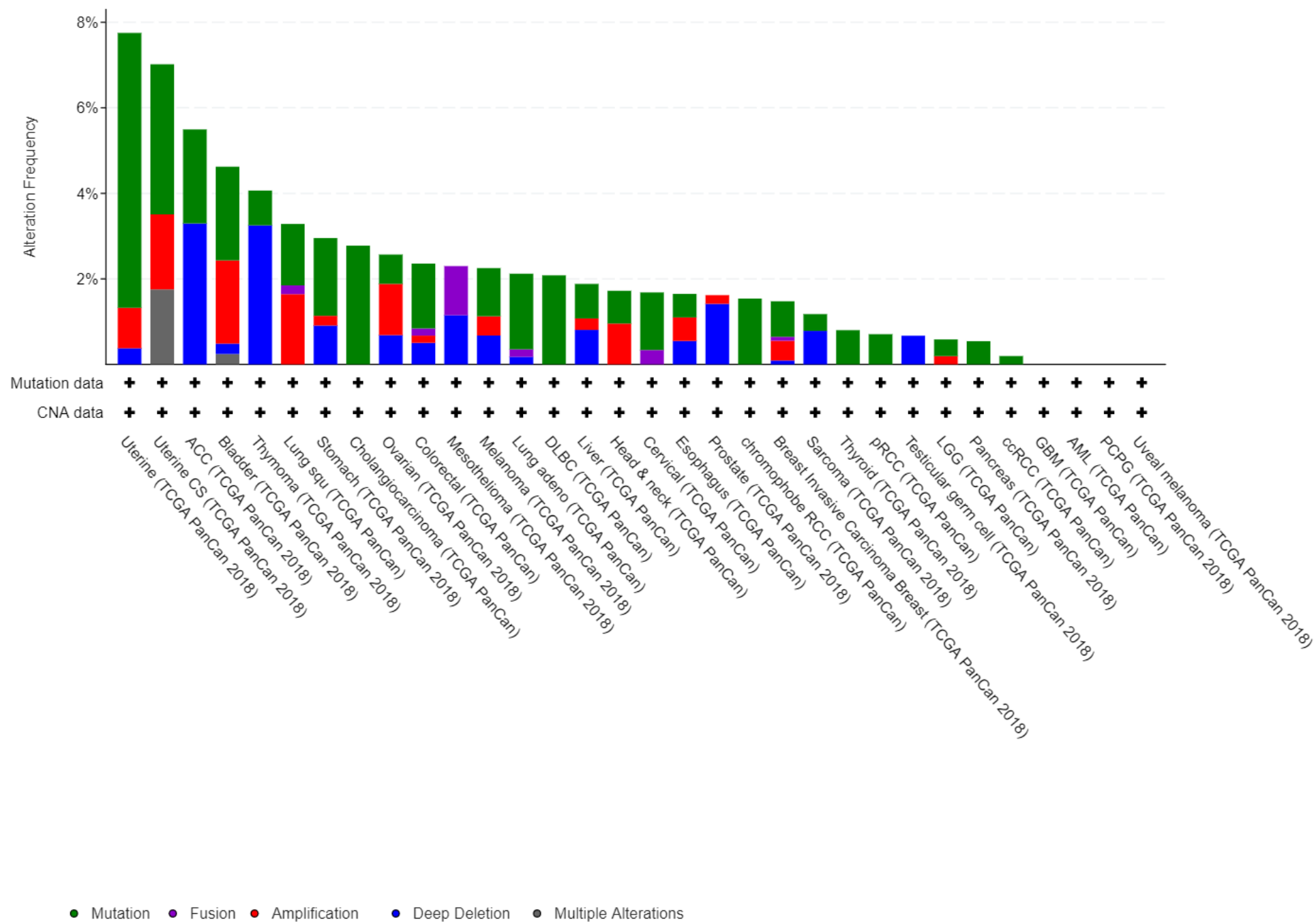

Figure S5,  
Wee1

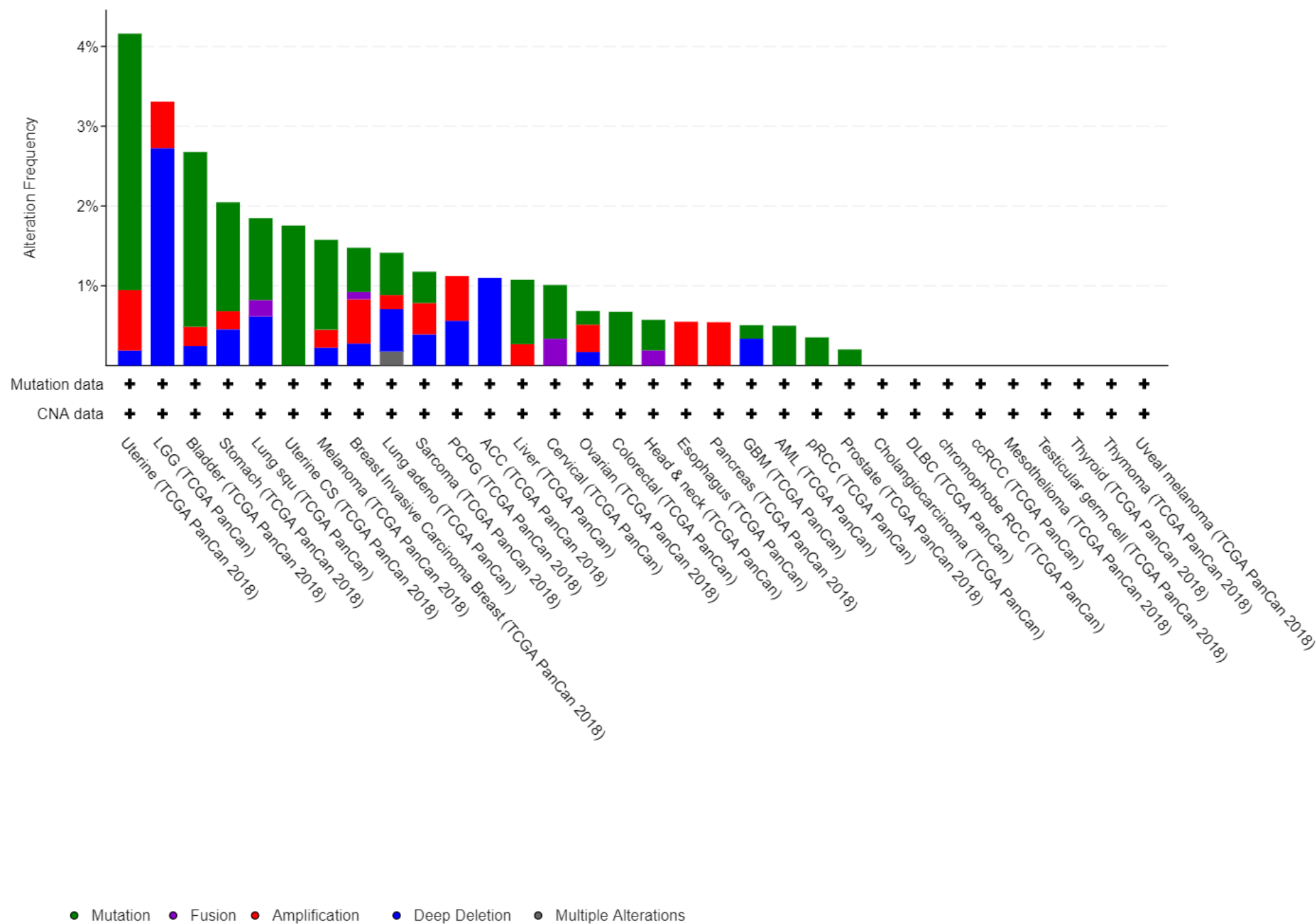

Supplement: Supplementary file 1 [file DataSheet_1.pdf]
